# The Anti-Inflammatory Role of Mannich Curcuminoids; Special Focus on Colitis

**DOI:** 10.3390/molecules24081546

**Published:** 2019-04-19

**Authors:** Gábor J. Szebeni, Lajos I. Nagy, Anikó Berkó, Alexandra Hoffmann, Liliána Z. Fehér, Mária Bagyánszki, Beáta Kari, József A. Balog, László Hackler, Iván Kanizsai, Anikó Pósa, Csaba Varga, László G. Puskás

**Affiliations:** 1 Laboratory of Functional Genomics, Biological Research Centre, Hungarian Academy of Sciences, Temesvári krt. 62, H-6726 Szeged, Hungary; g.szebeni@avidinbiotech.com (G.J.S.); balog.jozsef@brc.mta.hu (J.A.B.); 2 Department of Physiology, Anatomy and Neuroscience, Faculty of Science and Informatics, University of Szeged, Közép fasor 52, H-6726 Szeged, Hungary; berko@bio.u-szeged.hu (A.B.); hoffmannalexandra1228@gmail.com (A.H.); bmarcsi@bio.u-szeged.hu (M.B.); vacs@bio.u-szeged.hu (C.V.); 3 Avidin Ltd., Alsó kikötő sor 11/D, H-6726 Szeged, Hungary; l.nagy@avidinbiotech.com (L.I.N.); l.feher@avidinbiotech.com (L.Z.F.); b.kari@avidinbiotech.com (B.K.); i.kanizsai@avidinbiotech.com (I.K.); 4 AstridBio Technologies Ltd., Alsó kikötő sor 11/D, H-6726 Szeged, Hungary; hackler@avidinbiotech.com; 5 Department of Physiology, Anatomy and Neuroscience, Interdisciplinary Excellence Centre, Faculty of Science and Informatics, University of Szeged, Közép fasor 52, H-6726 Szeged, Hungary; paniko@bio.u-szeged.hu

**Keywords:** curcumin, Mannich curcuminoids, inflammation, colitis, inflammatory bowel disease

## Abstract

The incidence of inflammatory bowel disease (IBD) increases gradually in Western countries with high need for novel therapeutic interventions. Mannich curcuminoids, C142 or C150 synthetized in our laboratory, have been tested for anti-inflammatory activity in a rat model of TNBS (2,4,6-trinitrobenzenesulphonic acid) induced colitis. Treatment with C142 or C150 reduced leukocyte infiltration to the submucosa and muscular propria of the inflamed gut. C142 or C150 rescued the loss of body weight and C150 decreased the weight of standard colon preparations proportional with 20% less tissue oedema. Both C142 and C150 curcumin analogues caused 25% decrease in the severity of colonic inflammation and haemorrhagic lesion size. Colonic MPO (myeloperoxidase) enzyme activity as an indicator of intense neutrophil infiltration was 50% decreased either by C142 or C150 Mannich curcuminoids. Lipopolysaccharide (LPS) co-treatment with Mannich curcuminoids inhibited NF-κB (nuclear factor kappa B) activity on a concentration-dependent manner in an NF-κB-driven luciferase expressing reporter cell line. Co-treatment with LPS and curcuminoids, C142 or C150, resulted in NF-κB inhibition with 3.57 μM or 1.6 μM half maximal effective concentration (EC_50_) values, respectively. C150 exerted a profound inhibition of the expression of inflammatory cytokines, tumor necrosis factor-α (TNF-α), interleukin-6 (IL-6), and interleukin-4 (IL-4) in human PBMCs (peripheral blood mononuclear cells) upon LPS stimulus. Mannich curcuminoids reported herein possess a powerful anti-inflammatory activity.

## 1. Introduction

Acute inflammation is an evolutionarily advantageous primary response to infections or tissue injury. After the recruitment of inflammatory leukocytes and production of inflammatory mediators the resolution of the inflammation is a pre-requisite for physiological homeostasis. Chronic inflammation may be a consequence of a primary disease, like persistent infection, stroke, or heart disease, or the cause of a secondary disease, like autoimmune diseases, diabetes, Amyotrophic lateral sclerosis, Alzheimer’s disease, and even depression [1]. Smouldering chronic inflammation may be considered an individual disease without manifesting symptoms associated with a known disease [2]. Several molecular mechanisms link chronic inflammation with pathologies. Previously, we thoroughly reviewed cancer-related inflammation elsewhere [3,4]. The overall discussion of chronic inflammation-related pathologies is beyond the scope of the current paper. Our current work focuses on the effects of curcuminoids in ulcerative colitis. 

Inflammatory bowel disease (IBD) is a group of chronic inflammatory disorders of the gastrointestinal tract. Ulcerative colitis (UC) and Crohn’s disease (CD) are the two major subtypes of IBD. While they share common symptoms—chronic, idiopathic, relapsing, abdominal pain–there are clinical parameters and epidemiological features that are entirely different [5]. The main differences between UC and CD is the tissue localization of the disease. While inflammation persists in the mucosal and submucosal layers of the colon in UC, in cases of CD typically the entire gastrointestinal tract is affected. Incidence of both UC and CD is growing worldwide, with the highest annual incidence of UC, 24.3 per 100,000 person/years, and of CD, 12.7 per 100,000 person/years, in Europe [6]. While the pathogenesis of UC is still unknown, numerous theories have been proposed. It was suggested that interaction of immunological, environmental, and microbial factors contributes to the manifestation of the disease. We have previously shown the anti-inflammatory effect of physical exercise; recreation led to reduced area of lesions and decreased expression of Il-1β (interleukin 1 beta), CXCL1 (C-X-C motif chemokine ligand 1), and MPO in a rat model of colitis [7]. We have also reported the alleviated mucosal and neuronal damage in recurrent colitis [8] and the development of intestinal strictures due to high MMP9 (matrix metallopeptidase-9) and lower TIMP1 (TIMP metallopeptidase inhibitor 1) expression in the colon [9]. In the treatment of UC oral or intravenous steroids, calcineurin inhibitors (tacrolimus, cyclosporine) anti-inflammatory drugs, compounds containing 5-aminosalicylic acid, thiopurins and immunomodulators (e.g. tumor necrosis factor (TNF)-α targeting monoclonal antibodies), are administered [10,11]. Despite novel medical treatments, about 20–30% of UC patients need surgical intervention eventually [12]. Since the overall cure of UC was unresolved, our attention turned toward a dietary supplement, namely curcumin with anti-inflammatory behavior, to treat colonic inflammation [13]. 

Turmeric spice, curcuma, has been used as a dietary supplement for centuries for its intense yellow coloration and special taste in addition to its empirical beneficial health effects [14]. Curcuminoids, active components of turmeric, can be extracted from the rhizomes of Curcuma longa with different ratios: Dihidroxy-dimetoxycurcumin (curcumin, 75–77%), mono-demethoxycurcumin (17–19%), and bis-demethoxycurcumin (2–3%) [15]. Clinical trials verified the safety of curcumin intake without reporting toxicity dosing for up to 8000 mg daily for three months in patients with different malignancies [16]. Lao et al. treated healthy volunteers with high doses of up to 12000 mg of curcumin with 30% of the subjects reporting only grade 1 side effects [17]. Regarding the safety of curcumin, the Food and Drug Administration office of the USA has approved curcumin with a “generally regarded as safe” GRAS designation [18]. The anti-inflammatory properties of curcumin have been previously demonstrated [13]; however, its inherent poor bioavailability presents an obstacle for its clinical application [19]. In an effort to circumvent this problem, we have synthetized analogs of curcumin to increase their efficacy and pharmacodynamics. Our group previously reported the anticancer effect of Mannich curcuminoids in lung cancer [20], leukemia [21], pancreas [15], and glioma [22] models. Here, we demonstrate, for the first time, the potent anti-inflammatory effect of Mannich curcuminoids in an induced colitis model in a rat. 

## 2. Results

### 2.1. Relation of Mannich Substrates to Naturally Occurring Curcuminoids

**Figure 1:**
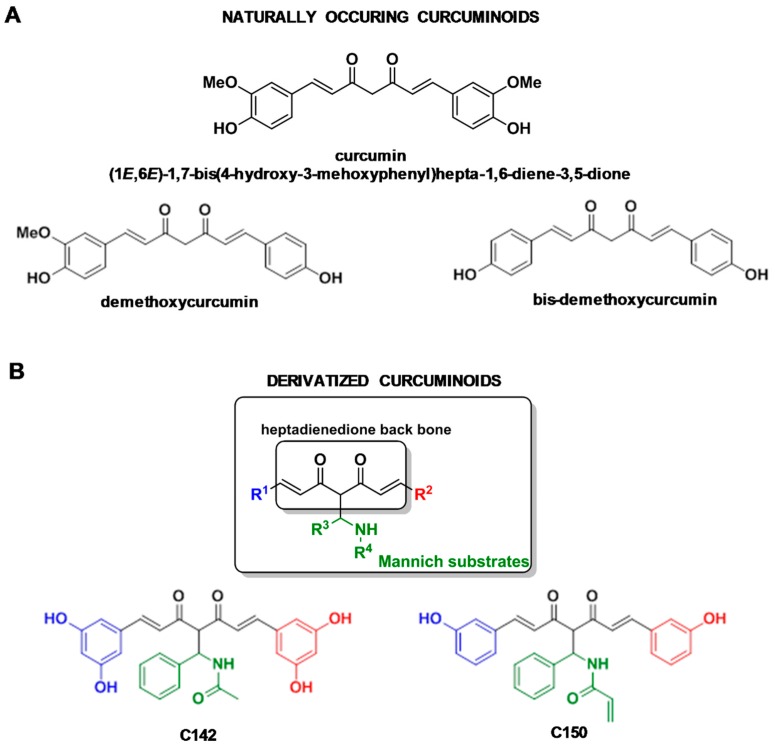
(**A**) Structures of naturally occurring curcuminoids and (**B**) structures of Mannich curcuminoids C142: *N*-((E)-5-(3,5-dihydroxyphenyl)-2-((E)-3-(3,5-dihydroxyphenyl)acryloyl)-3-oxo-1-phenylpent-4-en-1-yl)acetamide and C150: *N*-((E)-5-(3-hydroxyphenyl)-2-((E)-3-(3-hydroxyphenyl)acryloyl)-3-oxo-1-phenylpent-4-en-1 yl)acrylamide.

Recently, a library of a structurally diverse set of 47-membered Mannich curcuminoid was created via a powerful Mannich-3CR (M-3CR)/Claisen-Schmidt condensation sequence [20]. Utilization of the Mannich protocol, including the assemblies of aromatic aldehydes, primary amines, and pentane-2,4-dione, led to the formation of various Mannich structures as valuable intermediates with two optionally variable diversity points. Their treatment with aromatic aldehydes constituted a symmetrically substituted heptadienedione back-bone furnishing the desired curcuminoid species via morpholinium chloroacetate-catalyzed Claisen-Schmidt condensation [20,23]. Sythesis and NMR (nuclear magnetic resonance) analysis have been published earlier [20]. Here, we focus on the anti-inflammatory effect of derivatives of naturally occurring curcumin (Figure 1A) two Mannich curcuminoids C142: *N*-((E)-5-(3,5-dihydroxyphenyl)-2-((E)-3-(3,5-dihydroxyphenyl)acryloyl)-3-oxo-1-phenylpent-4-en-1-yl)acetamide and C150: *N*-((E)-5-(3-hydroxyphenyl)-2-((E)-3-(3-hydroxyphenyl)acryloyl)-3-oxo-1-phenylpent-4-en-1-yl)acrylamide (Figure 1B).

### 2.2. Anti-Inflammatory Effects of Curcumin Analogues in 2,4,6-trinitrobenzenesulphonic acid (TNBS) Induced Colitis

**Figure 2:**
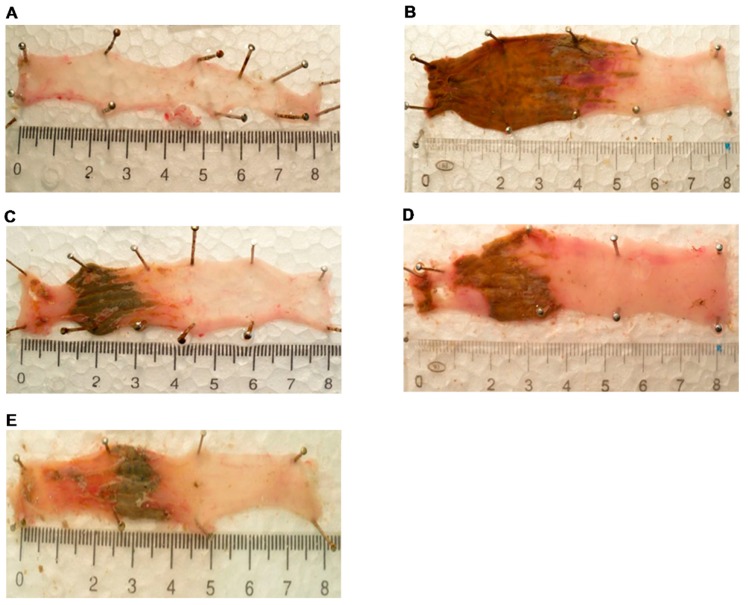
The 2,4,6-trinitrobenzenesulphonic acid (TNBS)-induced colitis developed to a macroscopic inflammatory damage in the colon of rats. Experimental groups were as follows: (**A**) Absolute control, (**B**) TNBS-induced colitis, (**C**) TNBS + C142, (**D**) TNBS + C150, and (**E**) TNBS + Infliximab. Representative images of 8 cm long fresh colon preparations after treatments. Colitis was induced as described in Section 4.2 in Materials and Methods.

Colonic administration of 2,4,6-trinitrobenzenesulphonic acid (TNBS) resulted in inflammatory damage of the colon inspected macroscopically 72 h after the challenge. The occuring macroscopic damage represented haemorrhagic necrosis due to intense tissue inflammation and hyperaemia (Figure 2B). Mannich curcuminoids, C142 and C150 (Figure 2C,D, respectively), exerted a protective effect at comparable degrees to the golden standard therapeutic, TNF-α neutralizing antibody, Infliximab (Figure 2E). There was no deleterious effect detected in the absolute control (vehicle-treated non-colitis-induced) group of rats (Figure 2A).

**Figure 3:**
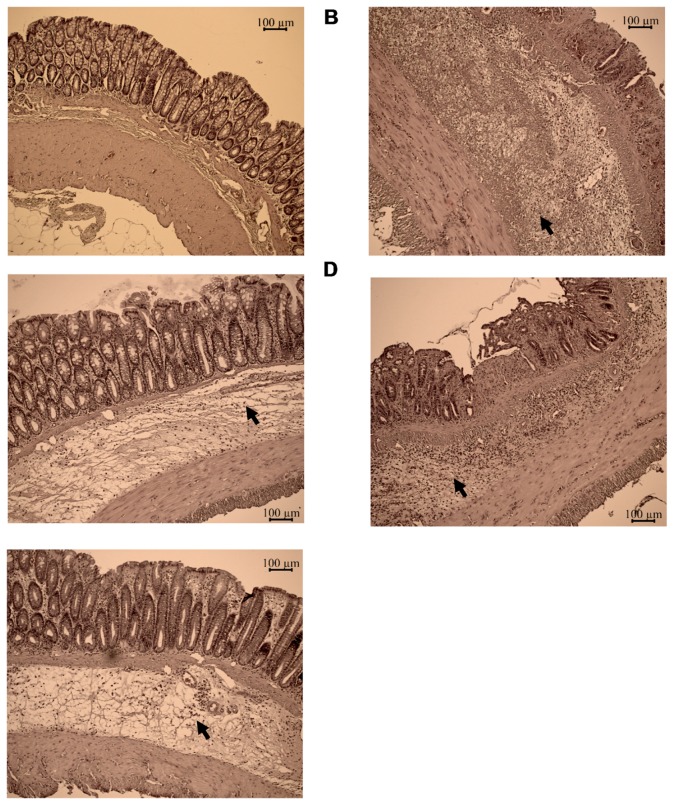
The TNBS-induced colitis developed to local inflammation (tissue oedema and infiltration of inflammatoy cells, arrowheads) in the colon. Experimental groups were as follows: (**A**) Absolute control, (**B**) TNBS-induced colitis, (**C**) TNBS + C142, (**D**) TNBS + C150, and (**E**) TNBS + Infliximab. Representative H and E images of colon preparations after treatments as described in Section 4.4 in Materials and Methods. Scale bar = 100 μm.

While healthy lamina propria and submucosa of the absolute control animals contained only few leukocytes (Figure 3A), histomorphology of the colon specimens revealed the local inflammatory tissue architecture and cellular composition of the rats bearing colitis (Figure 3B). Sever colitis represented an enlarged, oedematous submucosa with extended leukocyte infiltration even to muscularis propria (Figure 3B). These signs of acute colitis were milder in the treated animals by both C142 (Figure 3C), C150 (Figure 3D), or Infliximab (Figure 3E) showing reduced thickness of the submucosa and lower degrees of lymphoreticular infiltrate.

**Figure 4:**
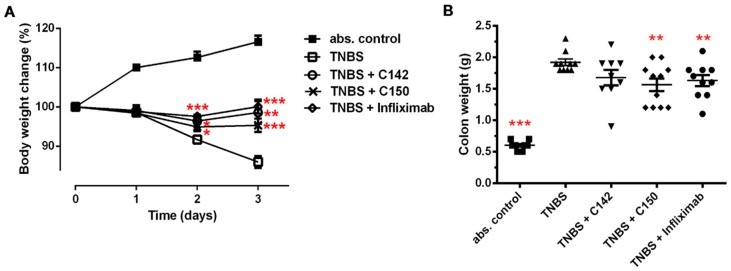
(**A**) Body weight change and (**B**) colon weight change in TNBS-induced colitis. Treatment with C142 or C150 rescued the loss of body weight and the degree of tissue oedema in the colon. Experimental design and treatments are described in Section 4.2 and Section 4.3 in Materials and Methods. Results are shown as mean ± S.E.M.; *n* = 8–11; * *p* < 0.05, ** *p* < 0.01, *** *p* < 0.001 pair-wise comparison with TNBS-treated group.

Initially, following TNBS treatment, animals showed symptoms of weakness, bloody diarrhea, and weight loss, while rats in the absolute control group gained weight (Figure 4A). By the end of the third day, rats with colitis lost 20% of their body weight, while controls increased their weight by 15%. Treatment either by curcuminoids (C142 *p* < 0.01, C150 *p* < 0.001) or Infliximab (*p* < 0.001) almost preserved the initial body weight of rats bearing colitis (Figure 4A). The weight of the standard colonic segment upon investigation corresponded to the degree of local colonic oedema [24]. Colonic instillation of TNBS resulted in four times higher colon weight compared to sham (only vehicle-treated) animals (*p* < 0.001). Similarly to Infliximab, C150, the acrylamid Mannich curcumin derivative inhibited the colonic oedema, reducing the effect of TNBS by almost 20% (*p* < 0.01) (Figure 4B).

**Figure 5:**
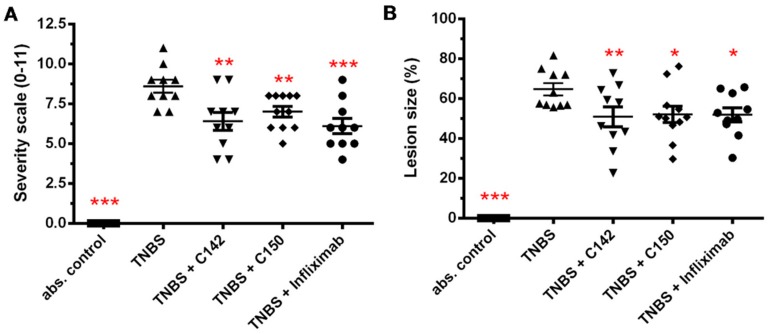
(**A**) Severity scale and (**B**) lesion size of the inflamed colon preparations. Both C142 and C150 curcumin analogues exerted a significant decrease in the severity of colonic inflammation and lesion size. Severity scaling and the measurement of lesion size was performed as described in Section 4.5 of Materials and Methods. Results are shown as mean ± S.E.M.; *n* = 8–11; * *p* < 0.05, ** *p* < 0.01, *** *p* < 0.001 pair-wise comparison with TNBS-treated group.

The severity of the colonic destruction was scored on a 0–11 scale in a randomized, blinded fashion according to Boughton-Smith et al. [25]. Scores directly correspond to the extent of inflammation and ulceration in the standard colonic segment. Challenge with TNBS resulted in an 8.6 ± 0.4 (*n* = 10) inflammatory damage score after 72 h (Figure 5A). Both C142 and C150 decreased the severity of macroscopic mucosal damage to 6.4 ± 0.56 (*p* < 0.01) and 7.0 ± 0.33 (*p* < 0.01), respectively (Figure 5A). Infliximab had the most potent anti-inflammatory effect, reducing severity to 6.1 ± 0.48 (*p* < 0.001) (Figure 5A). Planimetry was used to quantify the area of macroscopic lesions, the haemorrhagic and necrotic colonic areas expressed as a % of the total area under investigation. TNBS destructed 64.4 ± 2.98% of the standard colonic area, while treatments reduced macroscopic colonic damage by an average 20–25% (C142: 50.8 ± 5.05, *p* < 0.01; C150: 52.1 ± 4.06, *p* < 0.05 and Infliximab: 51.8 ± 3.49, *p* < 0.05) (Figure 5B). 

### 2.3. Effect of Mannich Curcuminoids on Inflammatory Mediators

**Figure 6:**
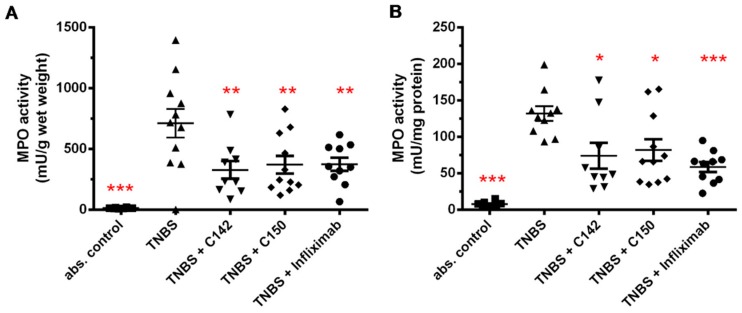
Effects of curcumin analogues on total tissue myeloperoxidase (MPO) activity (mU) normalized to (**A**) wet weight (grams) of the tissue or (**B**) protein content (mg) in the sample. MPO enzyme activity was significantly decreased both by C142 and C150 Mannich curcuminoids. MPO activity was measured as described in Section 4.6 of Materials and Methods. Results are shown as mean ± S.E.M.; *n* = 8–11; * *p* < 0.05, ** *p* < 0.01, *** *p* < 0.001 pair-wise comparison with TNBS-treated group.

Determination of myeloperoxidase (MPO) enzyme activity provides a measure of neutrophil infiltration to the inflamed colon [26]. We measured MPO activity from colon tissue homogenate following TNBS treatment and detected a dramatic increase of activity: 783.5 ± 103.5 vs. abs control 12.6 ± 1.6 mU/g wet weight (*p* < 0.001) (Figure 6A) and 131.7 ± 10 vs. abs control 7.5 ± 1.3 mU/mg protein (*p* < 0.001) (Figure 6B). Tested compounds decreased MPO activity to approximately 50 % of the TNBS group, suggesting lower infiltration of neutrophils to the inflamed colonic tissue, (C142: 326.7 ± 72.5, *p* < 0.01; C150: 370.5 ± 73.1, *p* < 0.01; Infliximab: 374 53.4, *p* < 0.01 mU/g wet weight (Figure 6A) and C142: 74.0 ± 17.8, *p* < 0.05; C150: 81.9 ± 14.7, *p* < 0.05; Infliximab 58.6 ± 6.9, *p* < 0.001 mU/mg protein (Figure 6B).

**Figure 7:**
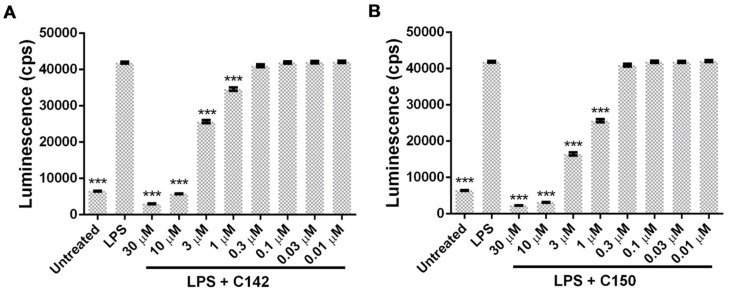
Mannich curcuminoids (**A**) C142 and (**B**) C150 inhibit lipopolysaccharide (LPS)-induced NF-κB activation in a concentration-dependent manner, as described in Section 4.7 of Materials and Methods. Luminescence (counts per seconds (cps)) is proportional to NF-κB-driven transcriptional activity. Results are shown as mean ± S.E.M.; *** *p* < 0.001 pair-wise comparison with LPS-treated group.

NF-κB, a master regulator of inflammatory conditions [27,28], plays a central role in the development of inflammatory cascade of the pathogenesis of colitis [29,30]. The involvement of NF-κB and whether Mannich curcuminoids could inhibit NF-κB-driven transcriptional activity have been investigated in an NF-κB-driven luciferase reporter cell line. Lipopolysaccharide (LPS), a cell wall component of Gram-negative bacteria, the canonical ligand of TLR4 (Toll-like receptor 4) receptor, has been reported to rapidly activate NF-κB [31]. LPS stimulation caused a five-fold induction of the reporter construct, while co-treatment with Mannich curcuminoids inhibited NF-κB activity in a concentration-dependent manner (Figure 7A,B). Co-treatment of LPS with C142 or C150, resulted in 3.57 μM or 1.6 μM EC_50_ values, respectively.

**Figure 8:**
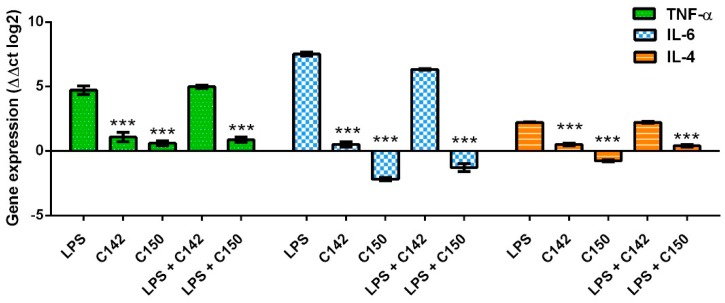
C150 inhibits the expression of tumor necrosis factor (TNF)-α, interleukin (IL)-6, and IL-4 upon LPS induction of human peripheral blood mononuclear cells (PBMCs) as described in Section 4.8 of Materials and Methods. Results are shown as mean ± S.E.M.; *** *p* < 0.001 pair-wise comparison with LPS-treated group.

During the pro-inflammatory response of colitis among others, NF-κB drives the expression of TNF-α and IL-6 [32,33]. Since TNF-α is a key factor in the acute phase response, its blocking by neutralizing antibodies reached a breakthrough in the management of colitis [34]. Similarly, IL-6 is also a therapeutic target in the clinical management of colitis [35]. Since our curcuminoids were able to inhibit NF-κB induced transcriptional activity we speculated that the expression of its downstream targets would also be inhibited. Another cytokine, IL-4 could be induced by LPS and could play a pro-inflammatory role in the intestinal microenvironment [36,37]. Therefore TNF-α, IL-6, and IL-4 gene expression was investigated in LPS-stimulated human peripheral blood mononuclear cells (PBMCs). Interestingly, qRT-PCR (quantitative real-time polymerase chain reaction) data revealed that only C150 exerted a profound inhibition of the expression of TNF-α (*p* < 0.001), IL-6 (*p* < 0.001), and IL-4 (*p* < 0.001) upon LPS stimulus (Figure 8).

## 3. Discussion

The incidence of inflammatory bowel disease (IBD) increases gradually in western countries and, despite the emerging field of anti-inflammatory drug development, there is a high need for novel therapeutics. Over the last few decades, several studies reported the therapeutic effect of naturally-occurring curcumin in colitis via scavenging free radicals, supporting antioxidants, inhibiting NF-κB, and myeloperoxidase activity, suppressing the production of inflammatory cytokines/chemokines (TNF-α, IL-6, interferon-γ, C-C motif chemokine ligand 17, chemokine (C-X-C motif) ligand 15) [38,39,40]. Since curcumin is a hydrophobic polyphenol with reduced solubility and stability in water-based solvents, which results in inefficient bowel absorption, low serum concentration, and consequently limited biological availability [41,42,43], it is necessary to design and synthetize curcumin analogs to increase bioavailability and potency [15,20,21,22]. 

Mannich curcuminoids, synthetized in our laboratory (Figure 1B), were tested for anti-inflammatory activity in a rat model of TNBS-induced colitis. The appearance of haemorrhagic and necrotic lesions was obvious after the 72 h induction of colitis in the 8 cm long standard colonic preparation under investigation (Figure 2B). Both C142 and C150 reduced the macroscopically-visible tissue destruction (Figure 2C,D) and the inflammatory conditions of the submucosal layers (Figure 3C,D). Treatment with C142 or C150 rescued the loss of body weight (Figure 4A) and C150 decreased the weight of standard colon preparations proportional to 20% less tissue oedema (Figure 4B). C142 and C150 curcumin analogues caused 25% decrease in the severity of colonic inflammation (Figure 5A) and haemorrhagic lesion size (Figure 5B). We investigated NF-κB activity, the master regulator of the development of inflammation. Lipopolysaccharide co-treatment with Mannich curcuminoids inhibited NF-κB activity in a concentration-dependent manner in an NF-κB-driven luciferase expressing reporter cell line (Figure 7). We also investigated the anti-inflammatory effect of Mannich curcuminoids on the activity of NF-κB target MPO and on the expression of NF-κB target cytokines TNF-α and IL-6 [44]. Colonic MPO enzyme activity used as a measure of intense neutrophil infiltration was 50% decreased by both C142 and C150 Mannich curcuminoids (Figure 6). C150 exerted a profound inhibition of the expression of cytokines: TNF-α, IL-6, and IL-4 in human PBMCs upon LPS stimulus (Figure 8). Mannich curcuminoids reported herein executed a powerful anti-inflammatory activity that provides the basis for further studies related to curcumin and curcumin analogues to treat colitis.

## 4. Materials and Methods

### 4.1. Animals 

Adult male Wistar rats (180–220 g) were kept in small groups. The animals had access to standard laboratory chow (Bioplan Ltd., Hungary) and drinking water ad libitum throughout the experiments. Rats were housed in a Minimal Disease animal house with controlled temperature (23 °C) and a light/dark (12 h:12 h) cycle. Before induction of colitis, food was withdrawn overnight and rats were placed in clean cages. The animal care and research protocols were in accordance with the national and international guidelines, reviewed and approved by the Regional Animal Health Authority, Csongrad County, in possession of an ethical clearance XX./592/2018. 

### 4.2. Experimental Design

Animals were divided into five groups randomly: 1, absolute control (vehicle treated non-colitis-induced, *n* = 8); 2, TNBS (vehicle treated colitis-induced, *n* = 10); 3, positive control (Infliximab treated colitis-induced, *n* = 10); 4, C142 curcumin analogue challenged (C142 treated colitis-induced, *n* = 10); and 5, C150 curcumin analogue challenged (C150 treated colitis-induced, *n* = 11). Before the beginning of the experimental period, rats were allowed to acclimate to the cages for a week, after that, colitis was induced by 2,4,6-trinitrobenzenesulphonic acid (TNBS, Sigma-Aldrich) once 10 mg was dissolved in 0.25 mL of 50 % (*w*/*v*) ethanol. The intracolonic administration of TNBS was performed with an 8 cm long plastic catheter under transient ether anesthesia, as described by Morris et al. [45]. Rats were weighed three times a week until the induction of colitis and following TNBS challenge, daily. Animals were sacrificed 72 h after the induction of colitis. The distal 8-cm long section of the colon was dissected, longitudinally opened, gently rinsed with ice-cold physiological saline, photographed (Panasonic lumix DMC-TZ6, digital camera, Kadoma, Japan) or the determination of macroscopic colonic inflammatory damage. The colon was weighed and divided for histology and into longitudinal segments to be used for the biochemical analyses. 

### 4.3. Dosage and Treatment

Each treatment started after colitis induction as soon as possible and was performed twice a day (at 9 am and 5 pm) during the experiment. Both curcumin analogues C142 and C150 (Avidin Ltd., Hungary) were administered orally in 40 mg/kg dosage. Common vehicle of curcumin analogues consists of 80% Peg-200 (Sigma-Aldrich, St. Louis., MO, USA) and 20% Chremophor EL (BASF, Ludwigshafen, Germany) dissolved in water for injection in 1:10 final ratio. The anti-TNF-α chimeric monoclonal antibody, Infliximab (Remicade, 100 mg, Schering-Plough, Kenilworth, NJ, USA), was administered once daily, intravenously in a 3 mg/kg dosage. Absolute and TNBS controls were treated with the vehicle of curcumin analogues orally. In order to treat the cells in culture curcumin analogues: C142 and C150 were dissolved at 10 mM in DMSO (Sigma-Aldrich) and further diluted in RPMI cell culture media (Thermo Fisher Scientific, Waltham, MA, USA).

### 4.4. Haematoxylin and Eosin Staining

Tissue samples for histology were taken 2–3 cm from the anus and fixed in 4% formaldehyde at 4 °C overnight. Tissue was placed into histology cassettes and dehydrated through graded ethanol to xylene and embedded in paraffin in a Leica TP1020 (Wetzlar, Germany) tissue processor. Sections were cut transversely at 5 μm thickness and processed for standard haematoxylin and eosin staining with Leica ST5010 Autostainer. Following staining, the tissue was mounted using a permanent mounting medium (CV Ultra, Leica). Sections were observed and photographed with a Zeiss Axio Imager.Z2 light microscope equipped with a Digital Microscopy Camera AxioCam ICc 5 (Oberkochen, Germany).

### 4.5. Damage Score and Lesion Measurement

The degree of colonic inflammation and ulceration was scored on a 0–11 scale in a randomized, blinded fashion adapted from Boughton-Smith et al. [25]. The criteria have been determined as described previously: 0 = no damage, 1 = focal hyperemia without ulcers, 2 = ulceration without hyperemia or bowel wall thickening, 3 = ulceration with inflammation at 1 site, 4 = more than 2 sites of ulceration and inflammation, 5 = more than 2 major sites of ulceration and inflammation or 1 site of ulceration/inflammation extending >1 cm along the length of the colon, 6–11 = the score is increased by 1 for each additional centimeter of involvement [24]. 

The extent of macroscopically-apparent inflammation, ulceration, and tissue disruption was determined in a randomized manner from the color images using proprietary computerized planimetry Java plugin (Stat_2_1_1, developed in our laboratory [7]) for ImageJ 1.0 (National Institutes of Health, the Laboratory for Optical and Computational Instrumentation (University of Wisconsin, Wisconsin, WI, USA). The area of macroscopically-visible mucosal involvement was calculated and expressed as the percentage of the total colonic segment area under investigation.

### 4.6. Measurement of Myeloperoxidase Activity

To measure myeloperoxidase activity we used a modified method described by Bradley et al. [46]. The 8 cm longitudinal strips of the colon were weighed, homogenized freshly (Ultra-turrax T25 IKA-Labortechnik, Staufen, Germany, 12,000 g, twice for 30 s; 250 mg colon/mL buffer) in ice-cold phosphate buffer (50 mM, pH 6.0), containing 0.5% CTAB (hexadecyltrimethylammonium bromide, Sigma-Aldrich), freeze-thawed three times in liquid nitrogen and then centrifuged at 10.000 g for 15 min at 4 °C. A 12 μL aliquot of the supernatant was then mixed with a 280 μL phosphate buffer (50 mM, pH 6.0), supplemented with 0.167 mg/mL O-adenosine dihydrochloride (Sigma-Aldrich), and the reaction was started with 10 µl 0.03% hydrogen peroxide, then assayed spectrophotometrically at 490 nm after 90 s shaking (Benchmark Microplate reader, Bio-Rad Labs, Hercules, CA, USA). MPO activity was expressed as mU/g wet weight tissue or mU/mg protein. A standard curve was created using serial dilution of peroxidase (Sigma-Aldrich). According to Bradley et al., one unit of the peroxidase activity was defined as that degrading one μmole of peroxide per minute at 25 °C [46].

In order to measure the protein concentration aliquots (20 μL) of the diluted protein, samples were mixed with 980 μL of distilled water. Bradford reagents (Sigma-Aldrich) (200 μL) were added to each sample and mixed vigorously. Following 10 min incubation, the samples were assayed spectrophotometrically at 595 nm. Protein level was expressed as mg protein/mL.

### 4.7. NF-κB Inhibition Assay

Mouse B16 melanoma cell line was purchased from the American Type Culture Collection (ATCC, Manassas, WV, USA) and was cultured in RPMI medium supplemented with 10% Fetal Calf Serum (FCS, Thermo Fisher Scientific). NF-κB reporter B16 cell line was created by transfection with the pNF-κB-Luc/neo reporter construct with the FuGene reagent (Promega, Madison, Wisconsin, USA). Stable transfected cell lines were selected by Neomycin (G418, Sigma-Aldrich) treatment. B16/NF-κB-Luc cells (7 × 10^4^ cells/well) were grown on 96 well luminoplates (Corning Life Sciences, Corning, NY, USA). After overnight incubation, when cells reached confluency, cell culture medium was changed and cells were pre-treated with a serial dilution of C142 and C150 curcumin analogs (or vehicle control) for 30 min before induction of NF-κB by lipopolysaccharide (LPS, Escherichia coli O111:B4, 500 ng/mL, Sigma-Aldrich). After 6 h of incubation the cell culture medium was removed and the cells were washed with phosphate-buffered saline (PBS) and lysed for 10 min at room temperature in Luciferase Cell Culture Lysis Reagent (20 μL/well, Promega). Substrate (luciferine) was added (20 μL/well, Bright-Glo™ Luciferase Assay System, Promega) and luciferase activity (counts per seconds (cps)) was measured in a microplate reader (Victor, Perkin Elmer, Waltham, MA, USA). NF-κB inhibition was calculated with relation to vehicle control cells from nine parallel measurements for all treatment conditions. The half-maximal effective concentration (EC_50_) was determined in relation to only LPS-treated wells in GraphPad Prism (version 5.01, La Jolla, CA, USA).

### 4.8. Quantitative Real-Time Polymerase Chain Reaction (qRT-PCR)

Human peripheral blood mononuclear cells (PBMC) were isolated from healthy volunteers by Ficoll density gradient centrifugation. Briefly, the withdrawal of 20 mL blood was carried out by a nurse into EDTA Vacutainer (Becton Dickinson, Franklin Lakes, NJ, USA). Twenty mL phosphate-buffered saline (PBS) was added to the blood under a safety laminar hood (Euroclone, Pero, MI, Italy). Leucosep tubes (Greiner Bio-One, Kremsmünster, Austria) were loaded with 20 mL diluted blood and centrifuged at 800 g for 20 min. After the removal of the plasma, the ring of PBMCs were collected into 15 mL conical tubes (Corning Life Sciences) and washed with 10 mL PBS at 500 g for 5 min two times. Cells were suspended in RPMI 10% FCS cell culture media and counted in a Bürker-chamber with trypan blue (Sigma-Aldrich). PBMCs (3 × 10^6^) were plated in 6 well tissue culture dishes (Corning Life Sciences) in RPMI 10% FCS. Cells were treated in 2 mL total volume with test compounds C142 and C150 at 2.5 μM or LPS at 100 ng/mL for 4 h. 

After treatment, nucleic acid preparation was done by using the RNA purification kit (Direct-zol^TM^ RNA MiniPrep Kit, Zymo Research, Irvine, CA, USA), according to an already published protocol [47]. The quality and quantity of the isolated RNA were measured with NanoDrop1000 Version 3.8.1. (Thermo Fisher Scientific). Reverse transcription from 600 ng of total RNA was performed with the High Capacity cDNA Archive Kit (Applied Biosystems, Foster, CA, USA) in a total volume of 30 μL according to the manufacturer’s protocol. Quantitative real-time PCR (qRT-PCR) was performed on the LightCycler^®^ 96 System (Roche, Basel, Switzerland), using gene-specific primers with SYBR Green protocol, as described previously [48]. Briefly, for cycling, each 10 μL PCR reaction contained 1 μL cDNA (20 ng), 250 nM primers, and 5 μL qPCRBIO SyGreen Mix Lo-ROX (2X, PCR Byosystems, London, UK). Primer sequences and accession numbers were as follows: IL6 (NM_000600.4; NM_001318095.1, F: 5’-GACCCAACCACAAATGCCAG-3’, R: 5’-GTGCCCATGCTACATTTGCC-3’); TNF (NM_000594.3, F: 5’-CACAGTGAAGTGCTGGCAAC-3’, R: 5’-GATCAAAGCTGTAGGCCCCA-3’); IL4 (NM_000589.4, NM_172348.2, NM_001354990.1) F: 5’- CCACGGACACAAGTGCGATA-3’, R: 5’- CTTCTGCTCTGTGAGGCTGTT-3’); ACTB (NM_001101, F: 5’-CCAACCGCGAGAAGATGA-3’, R: 5’-CCAACCGCGAGAAGATGA-3’. The PCR protocol was as follows: Enzyme activation at 95 °C for 2 min, 45 cycles of denaturation at 95 °C for 10 s, annealing at 60 °C, and extension at 60 °C for 10 s. All the qPCRs were performed with three replicates. After amplification, the melting curve was checked to verify the specificity of the PCR reactions. The Ct values were normalized to ACTB gene for each time point. The presented relative gene expression ratios were ΔΔCT values (log2). All values were presented as mean ± standard error of the mean (S.E.M.).

### 4.9. Data Representation and Statistical Analysis

Results are shown as mean ± S.E.M.; statistical comparisons were performed by two-tailed Student’s t-test as pairwise comparisons as described in the figure legends. In all statistical comparisons, probability “*p*” was set as the level of significance (set at * *p* < 0.05, ** *p* < 0.01, ***, *p* < 0.001). Data were processed and analyzed using Microsoft Excel (Microsoft Office 2016, Redmond, WA, USA), and visualized using GraphPad Prism.

## References

[1] Chen L., Deng H., Cui H., Fang J., Zuo Z., Deng J., Li Y., Wang X., Zhao L. (2018). Inflammatory responses and inflammation-associated diseases in organs. Oncotarget.

[2] Hunter P. (2012). The inflammation theory of disease. The growing realization that chronic inflammation is crucial in many diseases opens new avenues for treatment. EMBO Rep..

[3] Szebeni G.J., Vizler C., Kitajka K., Puskas L.G. (2017). Inflammation and cancer: Extra- and intracellular determinants of tumor-associated macrophages as tumor promoters. Med. Inflamm..

[4] Szebeni G.J., Vizler C., Nagy L.I., Kitajka K., Puskas L.G. (2016). Pro-tumoral inflammatory myeloid cells as emerging therapeutic targets. Int. J. Mol. Sci..

[5] Bernstein C.N., Fried M., Krabshuis J.H., Cohen H., Eliakim R., Fedail S., Gearry R., Goh K.L., Hamid S., Khan A.G. (2010). World gastroenterology organization practice guidelines for the diagnosis and management of ibd in 2010. Inflamm. Bowel. Dis..

[6] Molodecky N.A., Soon I.S., Rabi D.M., Ghali W.A., Ferris M., Chernoff G., Benchimol E.I., Panaccione R., Ghosh S., Barkema H.W. (2012). Increasing incidence and prevalence of the inflammatory bowel diseases with time, based on systematic review. Gastroenterology.

[7] Szalai Z., Szasz A., Nagy I., Puskas L.G., Kupai K., Kiraly A., Berko A.M., Posa A., Strifler G., Barath Z. (2014). Anti-inflammatory effect of recreational exercise in tnbs-induced colitis in rats: Role of nos/ho/mpo system. Oxid. Med. Cell Longev..

[8] Talapka P., Nagy L.I., Pal A., Poles M.Z., Berko A., Bagyanszki M., Puskas L.G., Fekete E., Bodi N. (2014). Alleviated mucosal and neuronal damage in a rat model of crohn’s disease. World J. Gastroenterol..

[9] Talapka P., Berko A., Nagy L.I., Chandrakumar L., Bagyanszki M., Puskas L.G., Fekete E., Bodi N. (2016). Structural and molecular features of intestinal strictures in rats with crohn’s-like disease. World J. Gastroenterol..

[10] Ford A.C., Moayyedi P., Hanauer S.B. (2013). Ulcerative colitis. BMJ.

[11] Meier J., Sturm A. (2011). Current treatment of ulcerative colitis. World J. Gastroenterol..

[12] Ordas I., Eckmann L., Talamini M., Baumgart D.C., Sandborn W.J. (2012). Ulcerative colitis. Lancet.

[13] Basnet P., Skalko-Basnet N. (2011). Curcumin: An anti-inflammatory molecule from a curry spice on the path to cancer treatment. Molecules.

[14] Hewlings S.J., Kalman D.S. (2017). Curcumin: A review of its’ effects on human health. Foods.

[15] Szebeni G.J., Balazs A., Madarasz I., Pocz G., Ayaydin F., Kanizsai I., Fajka-Boja R., Alfoldi R., Hackler L., Puskas L.G. (2017). Achiral mannich-base curcumin analogs induce unfolded protein response and mitochondrial membrane depolarization in panc-1 cells. Int. J. Mol. Sci..

[16] Cheng A.L., Hsu C.H., Lin J.K., Hsu M.M., Ho Y.F., Shen T.S., Ko J.Y., Lin J.T., Lin B.R., Ming-Shiang W. (2001). Phase i clinical trial of curcumin, a chemopreventive agent, in patients with high-risk or pre-malignant lesions. Anticancer Res..

[17] Lao C.D., Ruffin M.T.t., Normolle D., Heath D.D., Murray S.I., Bailey J.M., Boggs M.E., Crowell J., Rock C.L., Brenner D.E. (2006). Dose escalation of a curcuminoid formulation. BMC Complement. Altern. Med..

[18] Gupta S.C., Patchva S., Aggarwal B.B. (2013). Therapeutic roles of curcumin: Lessons learned from clinical trials. AAPS J..

[19] Liu W., Zhai Y., Heng X., Che F.Y., Chen W., Sun D., Zhai G. (2016). Oral bioavailability of curcumin: Problems and advancements. J. Drug Target.

[20] Gyuris M., Hackler L., Nagy L.I., Alfoldi R., Redei E., Marton A., Vellai T., Farago N., Ozsvari B., Hetenyi A. (2017). Mannich curcuminoids as potent anticancer agents. Arch. Pharm. (Weinheim).

[21] Nagy L.I., Feher L.Z., Szebeni G.J., Gyuris M., Sipos P., Alfoldi R., Ozsvari B., Hackler L., Balazs A., Batar P. (2015). Curcumin and its analogue induce apoptosis in leukemia cells and have additive effects with bortezomib in cellular and xenograft models. Biomed. Res. Int..

[22] Hackler L., Ozsvari B., Gyuris M., Sipos P., Fabian G., Molnar E., Marton A., Farago N., Mihaly J., Nagy L.I. (2016). The curcumin analog c-150, influencing nf-kappab, upr and akt/notch pathways has potent anticancer activity in vitro and in vivo. PLoS ONE.

[23] Mao H., Wan J., Pan Y. (2009). Facile and diastereoselective synthesis of β-acetamido ketones and keto esters via direct mannich-type reaction. Tetrahedron.

[24] Horvath K., Varga C., Berko A., Posa A., Laszlo F., Whittle B.J. (2008). The involvement of heme oxygenase-1 activity in the therapeutic actions of 5-aminosalicylic acid in rat colitis. Eur. J. Pharmacol..

[25] Boughton-Smith N.K., Wallace J.L., Whittle B.J. (1988). Relationship between arachidonic acid metabolism, myeloperoxidase activity and leukocyte infiltration in a rat model of inflammatory bowel disease. Agents Act..

[26] Khan A.A., Alsahli M.A., Rahmani A.H. (2018). Myeloperoxidase as an active disease biomarker: Recent biochemical and pathological perspectives. Med. Sci..

[27] Zhang Q., Lenardo M.J., Baltimore D. (2017). 30 years of nf-kappa b: A blossoming of relevance to human pathobiology. Cell.

[28] Vlahopoulos S.A. (2017). Aberrant control of nf-kappab in cancer permits transcriptional and phenotypic plasticity, to curtail dependence on host tissue: Molecular mode. Cancer Biol. Med..

[29] Andresen L., Jorgensen V.L., Perner A., Hansen A., Eugen-Olsen J., Rask-Madsen J. (2005). Activation of nuclear factor kappab in colonic mucosa from patients with collagenous and ulcerative colitis. Gut.

[30] Atreya I., Atreya R., Neurath M.F. (2008). Nf-kappab in inflammatory bowel disease. J. Intern. Med..

[31] Wang X.Y., Quinn P.J. (2010). Endotoxins: Lipopolysaccharides of gram-negative bacteria. Endotoxins.

[32] Deree J., Martins J.O., Melbostad H., Loomis W.H., Coimbra R. (2008). Insights into the regulation of tnf-alpha production in human mononuclear cells: The effects of non-specific phosphodiesterase inhibition. Clinics.

[33] Libermann T.A., Baltimore D. (1990). Activation of interleukin-6 gene expression through the nf-kappa b transcription factor. Mol. Cell. Biol..

[34] Levin A.D., Wildenberg M.E., van den Brink G.R. (2016). Mechanism of action of anti-tnf therapy in inflammatory bowel disease. J. Crohns. Colitis.

[35] Parisinos C.A., Serghiou S., Katsoulis M., George M.J., Patel R.S., Hemingway H., Hingorani A.D. (2018). Variation in interleukin 6 receptor gene associates with risk of crohn’s disease and ulcerative colitis. Gastroenterology.

[36] Mukherjee S., Chen L.Y., Papadimos T.J., Huang S., Zuraw B.L., Pan Z.K. (2009). Lipopolysaccharide-driven th2 cytokine production in macrophages is regulated by both myd88 and tram. J. Biol. Chem..

[37] Van Kampen C., Gauldie J., Collins S.M. (2005). Proinflammatory properties of il-4 in the intestinal microenvironment. Am. J. Physiol. Gastrointest. Liver Physiol..

[38] Toden S., Theiss A.L., Wang X., Goel A. (2017). Essential turmeric oils enhance anti-inflammatory efficacy of curcumin in dextran sulfate sodium-induced colitis. Sci. Rep..

[39] Baliga M.S., Joseph N., Venkataranganna M.V., Saxena A., Ponemone V., Fayad R. (2012). Curcumin, an active component of turmeric in the prevention and treatment of ulcerative colitis: Preclinical and clinical observations. Food Funct..

[40] Jian Y.T., Mai G.F., Wang J.D., Zhang Y.L., Luo R.C., Fang Y.X. (2005). Preventive and therapeutic effects of nf-kappab inhibitor curcumin in rats colitis induced by trinitrobenzene sulfonic acid. World J. Gastroenterol..

[41] Wang Y.J., Pan M.H., Cheng A.L., Lin L.I., Ho Y.S., Hsieh C.Y., Lin J.K. (1997). Stability of curcumin in buffer solutions and characterization of its degradation products. J. Pharm. Biomed. Anal..

[42] Mondal S., Ghosh S., Moulik S.P. (2016). Stability of curcumin in different solvent and solution media: Uv-visible and steady-state fluorescence spectral study. J. Photochem. Photobiol. B.

[43] Rego-Filho F.G., de Araujo M.T., de Oliveira K.T., Bagnato V.S. (2014). Validation of photodynamic action via photobleaching of a new curcumin-based composite with enhanced water solubility. J. Fluoresc..

[44] Yang Y., Wu J., Wang J.K. (2016). A database and functional annotation of nf-kappa b target genes. Int. J. Clin. Exp. Med..

[45] Morris G.P., Beck P.L., Herridge M.S., Depew W.T., Szewczuk M.R., Wallace J.L. (1989). Hapten-induced model of chronic inflammation and ulceration in the rat colon. Gastroenterology.

[46] Bradley P.P., Priebat D.A., Christensen R.D., Rothstein G. (1982). Measurement of cutaneous inflammation: Estimation of neutrophil content with an enzyme marker. J. Investig. Dermatol..

[47] Szebeni G.J., Balog J.A., Demjen A., Alfoldi R., Vegi V.L., Feher L.Z., Man I., Kotogany E., Guban B., Batar P. (2018). Imidazo[1,2-b]pyrazole-7-carboxamides induce apoptosis in human leukemia cells at nanomolar concentrations. Molecules.

[48] Szebeni G.J., Tancos Z., Feher L.Z., Alfoldi R., Kobolak J., Dinnyes A., Puskas L.G. (2017). Real architecture for 3d tissue (raft) culture system improves viability and maintains insulin and glucagon production of mouse pancreatic islet cells. Cytotechnology.

